# How will new genetic technologies, such as gene editing, change reproductive decision-making? Views of high-risk couples

**DOI:** 10.1038/s41431-020-00706-8

**Published:** 2020-08-09

**Authors:** Ivy van Dijke, Phillis Lakeman, Inge B. Mathijssen, Mariëtte Goddijn, Martina C. Cornel, Lidewij Henneman

**Affiliations:** 1 Amsterdam UMC, Vrije Universiteit Amsterdam, Department of Clinical Genetics, section Community Genetics and Amsterdam Reproduction & Development research institute, PO Box 7057, 1007 MB Amsterdam, The Netherlands; 2 Amsterdam UMC, University of Amsterdam, Department of Clinical Genetics and Amsterdam Reproduction & Development research institute, 1105 AZ Amsterdam, The Netherlands; 3 Amsterdam UMC, University of Amsterdam, Center for Reproductive Medicine, Women’s and Children’s Hospital and Amsterdam Reproduction & Development research institute, 1105 AZ Amsterdam, The Netherlands

**Keywords:** Genetic testing, Genetic counselling, Genetic engineering

## Abstract

Couples at increased risk of having offspring with a specific genetic disorder who want to avoid having an affected child have several reproductive options including prenatal diagnosis (PND) and preimplantation genetic testing (PGT). In the future, non-invasive prenatal diagnosis (NIPD), germline gene editing (GGE) and somatic gene editing (SGE) might become available. This study explores if, and how, availability of new genetic technologies, including NIPD, GGE, SGE, would change reproductive decision-making of high-risk couples. In 2018, semi-structured interviews were conducted with 25 genetically at-risk couples. Couples previously had received genetic counselling for PND and PGT, and in most cases opted for (one of) these techniques, at one Dutch Clinical Genetics Center between 2013 and 2017. Considerations participants mentioned regarding the hypothetical use of NIPD, GGE and SGE, seem similar to considerations regarding PND and PGT and are reflected in underlying concepts. These include safety and burden for mother and child, and moral considerations. Couples generally favoured NIPD over PND as this would be safe and enables earlier diagnosis. Increased opportunities of having a ‘healthy’ embryo and less embryo disposal were considerations in favour of GGE. Some regarded GGE as unsafe and feared slippery slope scenarios. Couples were least favourable towards SGE compared to choosing for a genetic reproductive technology, because of the perceived burden for the affected offspring. With the possibly growing number of technological options, understanding high risk couples’ perspectives can assist in navigating the reproductive decision-making process. Counsellors should be prepared to counsel on more and complex reproductive options.

## Introduction

Currently there are two main reproductive options available to couples facing an increased risk of having offspring with a specific genetic disorder and who wish to avoid having an affected child: invasive prenatal diagnosis (PND) (chorionic villus sampling or amniocentesis), possibly followed by termination of pregnancy if the foetus is affected, and preimplantation genetic testing (PGT). PGT involves intracytoplasmic sperm injection (ICSI), genetic testing and transfer of unaffected embryos with a strongly reduced chance of having to consider pregnancy termination, but requires an intense treatment process [1]. In addition, when pregnancy is achieved after PGT, couples are generally still offered PND because of the low risk of misdiagnosis. Other reproductive options are: refraining from having (more) children, using donor sperm or donor oocytes, considering adoption, or accepting the risk. Reproductive decision-making for high-risk couples is complex and known to be influenced by several factors, including the severity of the condition [2–6**]**, experiences with the condition [1–4, 6, 7], having an affected child [3, 5, 8], attitudes towards pregnancy termination [1, 6**–**8], the desire to have genetically related children [1, 8**]**, and perceptions on new technologies and their characteristics (e.g. safety) [1, 3, 6, 7].

In recent years, genetic technologies have developed rapidly. One new reproductive genetic technology is non-invasive prenatal diagnosis (NIPD) using cell-free placental DNA. NIPD nowadays is increasingly available for the detection of monogenic disorders, for example cystic fibrosis (CF) [9]. Challenges in performing the procedure and the costs involved, however, still limits its application [10]. Compared to PND, NIPD can be performed earlier in pregnancy, at around 8 weeks gestational age, and has no procedure-related miscarriage risk [10]. However, if the NIPD result shows that the foetus is affected, the couple is nevertheless confronted with a decision regarding continuing the pregnancy or not and in some cases invasive diagnostic confirmation is recommended [11].

Another, not yet available, possible future reproductive technology is germline gene editing (GGE). Targeted DNA changes could be performed in either immature oocytes and sperm or in early stage embryos, hereby ‘repairing’ the known disease-causing variant [12]. Although some people argue that PGT is sufficient to fulfil the wish to have an unaffected child, there are cases where GGE is the only option for conceiving genetically-related non-affected offspring, for example when both partners have CF [13]. GGE is a heavily debated technology. Due to experiments without regulatory oversight, the call for a broad societal debate on the socio-ethical and legal implications of GGE has intensified [14]. Besides GGE, developments in somatic gene editing (SGE), as a therapy for patients, are promising, as has been shown for thalassemia and sickle cell disease [15, 16]. SGE is considered less controversial than GGE since the DNA edits are made in somatic cells after the birth of an affected child and will not be passed on to future generations. The possible (future) availability of SGE as a treatment option, as well as other improvements in therapy, might change the need for current reproductive technologies [17].

It is not yet clear whether, and how, the developments in new reproductive technologies such as NIPD and GGE will influence couples’ reproductive decisions. On the one hand, new reproductive options might increase autonomy for genetically at-risk couples, while on the other hand, these developments could also complicate the already complex decision-making process and it could be more difficult to make an informed decision [18]. Concerns about the rapid introduction of new emerging reproductive genetic technologies in clinical practice are being raised by experts [13, 19], and little is known about how high-risk couples would perceive these new options. It is important to explore high-risk couples’ perspectives alongside the technical developments, as these couples might represent the possible future users [20]. Couples’ perspectives can inform policy-making and in response to their needs, genetic counselling can be adjusted. This study explores the views and considerations of genetically high-risk couples for opting for or against the (possible) future available techniques NIPD and GGE, in relation to their current reproductive decisions. Additionally, the potential influence of the availability of SGE on couples’ reproductive decision-making is explored.

## Materials and methods

A qualitative semi-structured interview study with both or one partner of couples at high risk of having affected offspring with a genetic condition was conducted. Approval was obtained from the medical ethical committee of the Amsterdam University Medical Centers, location Academic Medical Center (AMC) (W18_054).

### Participants and recruitment

Purposive sampling was used. Sixty-three out of 556 high-risk couples that had been referred to the Department of Clinical Genetics of AMC for genetic counselling about PGT and PND between 2013 and 2017, were invited. AMC is one of four centres in the Netherlands that offers genetic counselling on PGT. Throughout the article the term PGT is used for both PGT-M (Monogenic) and PGT-SR (Structural Rearrangements). Couples attending these counselling sessions come from different regions in the Netherlands, mostly from the Western provinces. Couples were invited for the study via both email and regular mail by two clinical geneticists [P.L. and I.M.]. A total of 25 interviews (with a total of 36 participants; ten couples and fifteen individuals belonging to a couple) were conducted (response rate 40% (25/63)). Reasons for non-participation were not investigated. To include a range of couples, the selection was based on different inheritance patterns (autosomal dominant, autosomal recessive, X-linked or chromosomal imbalance) and having an affected child or not. The selected couples had received genetic counselling by five different counsellors in the AMC.

### Procedure and interview guide

The interviews, lasted around 45 min. Of the 25 interviews, 18 were conducted face-to-face and seven by telephone between April 2018 and July 2018. One researcher [I.D], experienced in qualitative research and who had not been involved in the previous genetic counselling of the couples, conducted all the interviews. All participants signed informed consent. An interview guide was developed by the research team consisting of a clinical geneticist, an embryologist, and two health scientists. The first part of the interview focused on factors that influenced reproductive decisions previously made. In the second part, the new (future) reproductive technologies NIPD and GGE were introduced and briefly explained by the interviewer, as well as SGE as a possible future available therapy (see Supplementary Appendix 1). It was explained that NIPD is the most developed technology. In addition GGE and SGE were explained as possible future (reproductive) genetic techniques in a hypothetical way.

### Data analysis

The interviews were audio-recorded and transcribed verbatim. For reporting and analysing qualitative data we adhered to the 32-item COREQ checklist [21]. Thematic content analysis with open coding and interviewing was performed simultaneously [22]. Interviewing stopped when data saturation was reached, i.e. no new themes were generated. For the analyses, the transcripts were read multiple times to get familiar with the data and to enhance validity. Six interviews were independently coded by L.H. and I.D. to increase reliability; all other transcripts were coded by I.D. In an iterative process, the codes were grouped into relevant themes and clustered into categories related to the research question. A preliminary codebook was drafted deductively and adjusted after the first transcripts were coded. The final codebook was established to ensure efficient analysis. Findings were discussed with three researchers [I.D.,L.H.,P.L.] until consensus was reached. ATLAS.ti. software was used to manage the data and coding process. Quotes were translated from Dutch to English to illustrate the themes.

## Results

Participants’ characteristics and reproductive decisions are summarized in Table 1. Of the 25 couples, six couples had refrained from PGT. Of the 19 couples that opted for PGT, five couples were still going through the PGT treatment process, while four couples had already completed the process and ten couples had stopped because of a spontaneous pregnancy or other reasons. Fourteen couples had opted for PND. Five couples had children that were affected with the genetic condition and/or were deceased. Four of the six participants who were a carrier of an autosomal dominant disorder suffered from symptoms of the condition themselves (Supplementary Table S1).

**Table 1:** Characteristics of participants, *n* = 35.

Characteristics	
Gender, *n* (%)	
Female	24 (68.6)
Male	11 (31.4)
Interviews, *n* (%)^a^	
Couples (woman and man present)	10 (40.0)
Individuals	15 (60.0)
Mean age in years (SD) [range]	33.2 (3.89) [27–40]
Dutch background, n (%)^b^	33 (94.3)
Education, *n* (%)^c^	
Low	–
Intermediate	9 (25.7)
High	26 (74.3)
Religious, *n* (%)^d^	6 (17.1)
Type of inheritance of condition at-risk, *n* (%)	
AD	6 (24.0)
AR	5 (20.0)
XLR/D	12 (48.0)
Chromosomal imbalance	2 (8.0)
Mean time between reproductive genetic counselling and interview in years (SD) [range]	3.4 (1.42) [1.0–6.0]

*AD* autosomal dominant: Andersen–Tawil syndrome, Gorlin syndrome, Hereditary breast and ovarian cancer (*n* = 2), Hereditary diffuse gastric cancer, Huntington’s disease. *AR* autosomal recessive: cystic fibrosis, Usher syndrome, Pontocerebellar hypoplasia type 2, congenital disorder of glycosylation type 1a, non-ketotic hyperglycinemia. *XLR* X-linked (recessive): adrenoleukodystrophy, Becker muscular dystrophy, chronic granulomatous disease, desmyopathy, Duchenne muscular dystrophy, Fabry disease, hemophilia, ornithine transcarbamoylase deficiency, Pelizaeus–Merzbacher disease. XLD, X-linked (Dominant): Fragile X syndrome (*n* = 3). Chromosomal imbalance: reciprocal translocation and inversion chromosome.

^a^25 interviews were conducted with a total of 35 participants, of which 10 were with couples and 15. were with individuals (belonging to an invited couple). Of the interviews with individuals, one was with a male and the others were with female participants.

^b^One participant was from India and one was from Spain.

^c^Low: primary school, lower level of secondary school, lower vocational training. Intermediate: higher level of secondary school, intermediate vocational training, High: high vocational training, university.

^d^Five participants considered themselves Christian but were not (or hardly) practicing 532and one participant was of another denomination.

The findings from the interviews are structured according to: (1) Couples’ previous considerations for opting for or refraining from PND and/or PGT; and (2) Perspectives on new reproductive technologies (NIPD, GGE) and SGE. Themes and representative quotes are presented in Tables 2 and 3, respectively.

**Table 2:** Couples’ considerations for and against PND and/or PGT illustrated with representative quotes.

Theme	Representative quote	Quote #
Safety for the unborn child and mother	“She [the counsellor] said that the risk of another miscarriage was too high and besides that, it’s not really pleasant for yourself as well [PND, invasive testing] […] So, I thought that was very scary and I was anxious about the test result.” [Couple, AR, #8]	1.1
	“I thought it was a horrible idea for such an instrument [needle with invasive testing] to be so close to my child. That is of course not very pleasant.” [Woman, XLR, #20]	1.2
Success rate and burden of the technique	“Yes, you will appear on the waiting list and then it would only be our turn somewhere mid-July. I think [PGT] is one big frustrating process. I cannot say otherwise.” [Man, AD, #2]	1.3
	“I always remember one comment, she [the counsellor] said: ‘Yes 30%, that’s of course not very much [for a pregnancy with PGT], but if you can win the lottery with 30% you would buy a lot of tickets’. Then I thought: yes, you can also see it that way…” [Woman, XLD, #21]	1.4
Decision-making regarding pregnancy termination	“Back then [previous pregnancy] we still had hope: it [fetus] is staying in the womb, but is it OK? You don’t dare to become attached to it. This time we wanted to avoid this [opted for PGT].” [Woman, chromosomal imbalance, #24]	1.5
	“I find it very difficult anyway, because if you are, after all, pregnant and then you hear: “Well, your child has a disorder,” then you have to make the decision: terminate yes or no. I think I will find that very, very, difficult” [Man, chromosomal imbalance, #25]	1.6
Preference for a natural pregnancy	“It’s simply no fun to make children this way [PGT]. Of course it’s not only about the sexual intercourse, but just the whole thing that you are not procreating naturally.” [Man, AD, #2]	1.7
Personal experiences with the genetic condition	“Well, that [PND] was not an option for us. Because I am here as well, you know.” [Woman affected with disorder, AD, #1]	1.8
	“That [opting for any technology; PND or PGT] would almost feel like a betrayal of my [affected] son; the one that I already have.” [Couple, AR, #9]	1.9

*AD* autosomal dominant, *AR* autosomal recessive, *PND* prenatal diagnosis, *PGT* preimplantation genetic testing, *XLD* X- linked (dominant), *XLR* X-linked (recessive).

**Table 3:** Couples’ perspectives on new (reproductive) technologies (NIPD, GGE and SGE) in relation to the existing reproductive options of PND and PGT: representative quotes by theme.

Theme	Representative quote	Quote #
*Perspectives on NIPD*		
Safety for the unborn child and mother	“… non-invasive, that sounds like music to our ears. You know that if you go into that amniotic sac and you are there where the baby is, you have a chance, yes, of infection, you name it. Yes [NIPD] always sounds better [than invasive testing].” [Woman, XLR, #19]	2.1
	“But when I look back, I think I had… yes, I think I had used it [NIPD], more to just be informed and that then, yes, you can already adjust to that news or something. Even if you do nothing with it [termination of pregnancy].” [Couple, AR, #7]	2.2
Earlier diagnosis and decision-making regarding termination of pregnancy	“… time is really everything. Of course, if you then terminate [after PND result], what do you terminate and at what stage is that [the foetus]. The curettage instead of giving birth makes a huge difference, but the number of weeks of waiting is…normally you tell people after 10–12 weeks that you are pregnant, if you already have the test result it is nice. Now, you have to stay quiet, but at a certain point it becomes physically complicated to do that.” [Women, XLD, #22]	2.3
Views towards NIPD compared to PGT	“I would not do that [terminate a pregnancy] for the reason that this gene would be in it, because if you have it [the mutation] that does not mean that you will be severely ill. There’s just a sensitivity to that cancer. And that means, yes, I can also live to be a hundred years old, because they got there early enough. But then yes, no, terminating a pregnancy is ethically crossing a line for me, availability of NIPD would not change the choice for PGT for me for this reason” [Women AD, # 1]	2.4
	“We have experienced through PGT that our number of affected embryos was so incredibly high. So I don’t know if I would dare [opt for NIPD]. I do think for us, PGT was a very safe way. Yes, of course, you would rather not terminate a pregnancy.” [Man, AD, #2]	2.5
*Perspectives on GGE*		
Safety concerns	“I would opt for PGT, because I have more faith in this, than to fix something [with GGE]. My car is sometimes repaired and it doesn’t always come back in good shape.” [Woman, XLR, #20]	3.1
More unaffected embryos and no embryo disposal compared to PGT	”But of course there are [more options] and certainly if you have multiple abnormalities. I mean, the more abnormalities, or chance of a disease, the nicer it is to cut and paste it a little, until its correct.” [Woman, XLD, #21]	3.2
	“Suppose it [GGE] is safe and the consequence is that you do not have to throw away embryos, so that you can have many more embryos from one puncture […] I have no moral objections to that. Surely it’s only beautiful if your child is not burdened with such an awful disease?” [Man, AD, #4]	3.3
Slippery slope scenarios and unnatural	“On the one hand, I find it [GGE] very scary, because you are messing with nature so much. But it would be great if it could be done like this. My gut feeling is that I’m against modifying DNA. If you could modify DNA, how far do you go? That’s also the ongoing discussion now […] You can choose your baby in a catalogue. I want red hair with freckles, white skin, very nice. A gene for breast cancer that can be detected very early, will you take it out or not? But glasses, yes, how bad are glasses?” [Woman, XLR, #15]	3.4
	“We are not religious or anything like that, but as far as I’m concerned […] I think with this you’re going a bit too far in human creation. Then you really intervene in the structure of the DNA, you intervene with nature.” [Woman, XLR, #19]	3.5
Costs and fears for inequality	“We can end up in such a world, that such differences are created, some can afford it [GGE] and others cannot. I don’t think that’s desirable.” [Man, AD, #2]	3.6
	“To fix it, let’s be honest, if you have sick children it costs society a lot of money. I see what our son with his illness costs with his illness […] It would be better for society if you could just bring healthy children into the world. It sounds a bit harsh […] If you know that you have a certain chance of having a sick child, I would say fix it.” [Woman, XLR, #20]	3.7
*Perspectives on SGE*		
Burden for the future child	“If you look at the options, then this [SGE] is the least interesting, because you are messing with a child that is still so small. They are still so vulnerable; I think that is difficult to watch. Look, what happens to me is not interesting, I don’t really care because I know what I do it for. But such a small person has no clue.” [Couple, AR, #8]	4.1
	“I think, let’s have all the fuss in advance. That will be on our account, the child is not bothered by it then […]. Of course it also depends on whether it all succeeds or not, or whether a complication occurs, and so on.” [Woman, AD, #3]	4.2
	“The advantage is a natural pregnancy, but I would also find it [SGE] very intense if such a small baby had to undergo enormously intense surgeries and that you know this beforehand…” [Couple, AR, #10]	4.3
Possibility of a natural pregnancy	“There were no issues in terms of fertility, so yes, the option of getting pregnant naturally is there [can opt for SGE], which I thought was a much nicer idea, but there is this risk [of having an affected child]…”[Woman, AD, #1]	4.4
	“Yes, because it [PGT] is a very heavy process. With this [SGE] you can get pregnant naturally. So yes, that sounds a lot easier to me… yes but what about the certainty [of SGE]?” [Woman XLR, #13]	4.5
	“Suppose you have completed the pregnancy, you have already furnished your house for the baby and then, the treatment would go wrong after all, then you already have that entire process of the entire pregnancy and gave birth and if you then have to say goodbye to your child, because that treatment does not work out well, that is terrible, I would opt for something else instead.” [Woman, chromosomal imbalance, #24]	4.6
Difficult to anticipate	“I don’t know, it’s [SGE] still very unknown. I have no idea, I find it hard. If your child is sick and it can be treated, everyone would opt for that. Even though it would take five surgeries until your child is healthy you would do it. Because you want your child to go through life without worries.” [Woman, XLR, #20]	4.7

*AD* autosomal dominant, *AR* autosomal recessive, *GGE* germline genome editing, *PND* prenatal diagnosis, *NIPD* non-invasive prenatal diagnosis, *PGT* preimplantation genetic testing, *SGE* somatic genome editing, *XLD* X- linked (dominant), *XLR* X-linked (recessive).

### Previous considerations for opting for or refraining from PND and/or PGT

Participants who initially opted for PND and/or PGT were all couples who chose these techniques because they preferred to avoid the birth of an affected child. They described their former process of reproductive decision-making as intense, complex and iterative. The initial reproductive option of choice was quite often not in concordance with the actual performed reproductive option, in particular regarding PGT (see Supplementary Table S1). For example, couples discontinued the (planned) PGT procedure because they had an (unexpected) spontaneous conception during the PGT preparation process, leaving PND as the only available option besides accepting the risk. Frequently mentioned considerations that influenced the decision regarding PND and/or PGT were: safety for the unborn child and mother because of the invasiveness of the procedure and fear for the procedure-related miscarriage risk with PND (Table 2, quote 1.1 and 1.2), the burden of the procedure and success rate of the technique, mostly mentioned in the context of PGT (quote 1.3 and 1.4), decision-making regarding pregnancy termination (quote 1.5 and 1.6), and the preference for a natural pregnancy (quote 1.7). Overarching factors, not necessarily related to the technologies, that influenced couples’ reproductive decision making were perceived severity and personal experiences with the disorder, such as having the condition themselves (in case of an autosomal dominant disorder) or having an affected child (quote 1.8. and 1.9).

### Perspectives on new (reproductive) genetic technologies

Considerations regarding NIPD, GGE and SGE were discussed in comparison to the available reproductive genetic technologies (PND and PGT) (Table 3). Generally, participants emphasized that they would only consider these new technologies if safety requirements are met, side effects are minimized, and success rates have been proven. The considerations in favour of and against these new technologies are summarized in Table 4.

**Table 4:** Considerations regarding the new technologies from the participants’ perspectives in terms of underlying concepts.

	Safety (for mother and child)	Burden (for mother and child)		Moral considerations	Natural pregnancy
*Considerations* in favour *of the new technologies*					
NIPD	Safe for the unborn child (compared to PND)	Less inconvenient for the mother (compared to PND)	Earlier testing (compared to PND): 1] Less time to become attached to the unborn child results in a less emotional decision regarding possible termination of pregnancy 2] Increased possibility of a medical abortion versus surgical or induced abortion procedure		Like in PND, with NIPD a natural pregnancy is a possibility which is experienced as a positive aspect compared with PGT
GGE		More possibilities – due to editing – of having unaffected embryos resulting in possibly less oocyte punctures (compared to PGT)		No embryo disposal (compared to PGT) Repairing embryos could save costs for individuals and society	
SGE		Having a ‘care-free’ pregnancy: no testing, no anxiety waiting for test results	When you (unexpectedly) have an affected child, this technique could offer a potential treatment for the child		Possibility of having a natural pregnancy without medical interventions (compared to PGT)
*Considerations* against *the new technologies*					
NIPD			If one wants to avoid an affected child, termination of pregnancy is still the only option which could be difficult when you morally object to termination		
GGE	Safety issues in terms of side effects and unintended consequences (compared to PGT)		Risk of slippery slope scenarios: how to decide on indications and how far do we go as a society? Fear for inequality in access Procedure is considered unnatural: ‘crossing a line’, modifying human DNA		
SGE	Safety concerns for the future child	‘Treatment’ burden for the future child (let parents carry the burden of reproductive technologies instead of the future child)	Prevention is better than cure (in order to make difficult decisions beforehand)		

*GGE* germline genome editing, *NIPD* non-invasive prenatal diagnosis, *PGT* preimplantation genetic testing, *PND* prenatal diagnosis, *SGE* somatic genome editing.

### Views towards NIPD

#### Safety for the unborn child and mother

If it would be possible to safely use NIPD for monogenic disorders, the responses of participants were generally positive. Participants considered the non-invasive component as the most appreciated characteristic of NIPD compared to PND (Table 3: quote 2.1). Some couples would want to use NIPD to be prepared and informed about the health of the unborn child instead of considering a pregnancy termination (quote 2.2).

#### Earlier diagnosis and decision-making regarding pregnancy termination

A diagnosis at an earlier gestational age was considered as an important advantage of NIPD compared to PND. A majority of the participants argued that the longer they are pregnant the more attached they become to the unborn child and termination becomes more difficult. Moreover, an earlier result would enable termination of pregnancy by curettage, instead of an induced early labour procedure, which was considered less traumatic (quote 2.3).

#### Views towards NIPD compared to PGT

Participants discussed NIPD in relation to PGT. Couples who wanted to avoid a possible decision regarding pregnancy termination would prefer PGT over either NIPD or PND. Terminating a pregnancy for a condition you suffer from yourself was considered a difficult decision by participants as illustrated in quotes 2.4 and 2.5. Some couples mentioned that they would prefer NIPD instead of PND during the PGT process, for confirmation of PGT results.

### Views towards GGE

#### Safety concerns

Overall participants seemed positive of the idea of GGE, but some were hesitant due to safety issue and side effects. They had more trust in PGT and mentioned that with GGE there was a chance that the induced DNA changes might have unintended consequences (quote 3.1), for example making the cut at the wrong place (known as off-target effects).

#### More unaffected embryos and no embryo disposal compared to PGT

The possibility of “repairing” affected embryos by GGE besides only selecting unaffected ones by PGT, was mentioned by participants as an opportunity to increase their chance of getting pregnant with an unaffected child, which was considered as a great advantage of GGE (quote 3.2). In addition, participants argued that women would have less unpleasant ovum pick-up procedures to undergo. Furthermore, discarding of embryos was mentioned by many participants as a negative aspect of PGT because of the moral status of embryos in general (quote 3.3). A few participants argued that with PGT their children might have to face the same reproductive dilemmas because, in their specific situation, the transferred embryos could be healthy carriers of the same disorder (e.g. selecting female embryos in X-linked disorders), which was another argument in favour of GGE.

#### Slippery slope scenarios and unnatural

Some participants feared that once GGE is allowed for treating severe genetic conditions in the embryonic stage, it would also be allowed for other goals, like enhancement purposes such as cosmetic changes and increased intelligence (quote 3.4). For some, gene editing of embryos was unnatural and interfering in the DNA is a line that should not be crossed (quote 3.5).

#### Costs and fears for inequality

Fears of abuse of the technology for the purpose of earning money or ambiguous indications were expressed. On the one hand concerns were voiced that only rich people would be able to afford GGE if health insurances would not reimburse it, giving rise to inequality issues (quote 3.6). On the other hand, participants mentioned that one of the expected benefits of GGE would be that repairing embryos might be cost-saving compared to destroying affected embryos (quote 3.7).

### Views towards SGE as a therapeutic option

#### Burden for the future child

Participants preferred to put the burden for decision making, for example undergoing the PGT process, on themselves over putting their child at risk for a possible available treatment (SGE). This resulted in a great reluctance regarding SGE (quote 4.1 and 4.2). Participants who were identified as a high-risk couple after the birth of an affected child, were more positive about SGE, but would not opt for SGE initially over other reproductive options because they expected it to be an intense burden for their future child (quote 4.3). Nevertheless, it reassured participants that there would be a treatment available if the PGT or PND test results turned out to be false negative. SGE was therefore viewed as a kind of “safety net”.

#### Possibility of a natural pregnancy

Once SGE would be proven safe and successful, some participants stated that they might opt for SGE and refrain from PND or PGT at all. Participants who preferred a natural pregnancy, with natural conception, and who therefore refrained from PGT, were generally more positive about SGE (quote 4.4 and 4.5) but also still cautious, as they feared that the therapy might not be successful (quote 4.6).

#### Difficult to anticipate

Most participants experienced a difficulty in forming a realistic opinion on SGE. Many found thinking in a hypothetical way about SGE as a future therapeutic option, without clear clinical examples, difficult to imagine (quote 4.7). Moreover, it could also feel like taking a risk because there is no information so far about the chance of treatment success. For this reason and for the possible burden on the future child several participants considered SGE the least favourable option to anticipate on.

## Discussion

This study explored the views of genetically high-risk couples, who previously received PND/PGT counseling, on NIPD, GGE and SGE in relation to the reproductive decisions they had previously made. Considerations participants mentioned regarding the hypothetical use of NIPD, GGE and SGE seem similar to considerations regarding PND and PGT and are reflected in underlying concepts, summarized in Table 4. These concerned the safety aspects and burden of the specific technologies, moral considerations such as attitudes towards the status of the embryo and pregnancy termination and the preference for a natural pregnancy.

As was shown in previous studies [18], participants were positive about NIPD because they considered it safer for mother and child and it enables earlier testing compared to PND. Therefore, a shift from current invasive prenatal methods to NIPD might possibly occur in the Netherlands. A negative attitude towards termination of pregnancy, however, was a reason to refrain from NIPD and to (still) opt for PGT as was shown previously [6], despite the favourable characteristics of NIPD. Some couples expressed negative attitudes towards pregnancy termination, but indicated that they would nevertheless opt for NIPD just to be informed and prepare for a child with a genetic condition, as was shown earlier for NIPD for CF [23] and non-invasive prenatal testing for Down syndrome [24]. Participants in our study expressed their views on NIPD in a different manner compared to experts as described in the literature. Experts generally emphasize technical aspects, as the labour-intensity and accuracy of NIPD [25], whereas participants from our study emphasized the attitudes towards pregnancy termination, earlier diagnosis, and the value of a natural pregnancy as important factors for consideration. It is needless to say that both valued safer diagnosis. Concerns of routinisation and informed consent were not expressed by participants, in contrast to a qualitative study in the United Kingdom (UK) with carriers of recessive disorders [26], in which issues around consent were explicitly addressed. Furthermore in the UK, in contrast to the situation in the Netherlands at the time of the interviews, NIPD is available in a growing number of hospitals, and favoured by high-risk couples [23], however challenges regarding costs remain [27].

GGE was considered generally positive because, according to participants, it would offer increased possibilities compared with PGT since more embryos, either unaffected or treated embryos, could be transferred and less embryos disposed [1], as previously stated by experts [19, 28]. In this way less IVF/ICSI cycles are needed, which could decrease burden for the mother. These findings are similar to previous studies assessing people’s perspectives [29, 30]. However, fears of the technology being abused were expressed. Couples reported concerns about the responsible use of GGE; it should be used only for serious conditions and not to create ‘designer babies’, as was shown by others [13, 19]. In the literature, it has been argued that availability of GGE might lead to discrimination of people who live with disabilities, as was discussed earlier for other selective reproductive technologies [31]. Changing the DNA of embryos was considered “a bridge too far” by some of the participants and therefore they would prefer the established PGT technology. Even though participants had concerns, they seemed generally more positive towards GGE than the general public, possibly because they reason from an experiential perspective [32]. However, a lack of in-depth understanding on the implications of genome editing (somatic as well as germline) could have biased participants’ views as the ability to provide arguments partly depends on prior knowledge [33].

Couples generally considered NIPD and GGE as options to anticipate on, provided these are safe, effective and available. Participants generally viewed taking the risk by conceiving naturally and treating an affected child after birth with SGE as the least favourable option to consider before pregnancy. Moreover, couples were uncertain about what SGE might look like clinically. This finding is in line with a US focus group and survey study that assessed the willingness of sickle cell patients to participate in SGE clinical trials [17]. In contrast to our findings, earlier studies among the general public were more supportive towards clinical use of SGE as opposed to GGE [30, 34]. Explanations for more public support of SGE could be that first, germline edits are inheritable and therefore considered controversial, while SGE could offer a curative treatment for individuals [29]. Second, in public opinion studies SGE was not discussed in relationship to reproductive technologies as in this study, but as a possible therapy. Clinical trials of SGE are ongoing and results are promising according to experts [35]. However, when offering SGE within a range of reproductive options (e.g. PGT or NIPD), participants are reluctant and would rather opt for reproductive options where the burden would be on the couples’ shoulders, instead of burdening their child with a treatment.

In addition to perspectives on the technologies explored in this study, other developments in the reproductive field should be taken into account, such as preconception carrier screening for recessive disorders, which may increase reproductive choice [36]. Moreover, trophoblast retrieval from the cervix (TRIC) could potentially detect foetal DNA at an even earlier gestational age compared to NIPD [37].

### Study strengths and limitations

This study has several strengths. Firstly, few studies have been conducted on possible future users’ views on new technologies such as GGE and SGE [38]. Secondly, a variety of participants were included in terms inheritance patterns to explore a broad range of perspectives. Limitations of this study are that, first of all participants were recruited from one centre only, which could have resulted in bias because they all received their genetic counselling regarding reproductive decision making in this centre. Moreover, participants mostly came from the Western provinces. As with the regional differences that are known for non-invasive prenatal testing(NIPT) uptake [39], this could influence people’s interest in other reproductive technologies and therefore results must be interpreted with caution. Healthcare systems and cultures differ between countries, and the perspectives of couples could as well [39]. Thirdly, only high-risk couples who primarily had expressed (some) interest in PGT, and who were counselled about PGT and PND, were interviewed. Couples who refrained from these options, and/or who refrained from having children in order to avoid an affected child, were not included in our study. Exploring their views will be a next step for research because they could have other perspectives on the technologies. Moreover, views on reproductive decision-making were explored retrospectively, which could have resulted in recall bias. Fourthly, participants were relatively highly educated, which could have biased the results [3]. Furthermore, seven interviews were conducted by telephone which limits body language response. Lastly, since GGE and SGE were explained in hypothetical scenarios, it was hard to elicit real perspectives that reflect the actual reproductive decision-making of the participants.

### Conclusion and implications

With the results of this study, bearing in mind that this is a study regarding the hypothetical use of future technologies, we attempted to gain more insight into the future dynamics of the reproductive decision-making process of high-risk couples counselled for PND and/or PGT who want to avoid the birth of an affected child. Understanding these couples’ perspectives can assist in navigating reproductive decision-making [7]. Genetic counsellors could bear in mind the concepts underlying decision-making identified in this study, and explore together with couples how they feel about the different options and support them in their decision. The non-invasiveness and earlier gestational diagnosis of NIPD are considered important advantages. Moreover when comparing PGT to NIPD results suggest that high-risk couples, who previously had made a reproductive decision, and who have objections towards termination of pregnancy will continue to opt for PGT, instead of opting for NIPD. The results may also suggest parallel use of GGE with PGT, if GGE is safe and effective, because of the potential larger number of embryos eligible for transfer. Opting for a natural pregnancy followed by treatment of the child after birth with SGE was evaluated as less positive compared to reproductive options before or during pregnancy, mainly because of the perceived burden for offspring. Though many of the couples’ considerations regarding these technologies remain the same in essence, with the growing number of reproductive options, genetic counselling will become more challenging as these new developments will most likely complicate reproductive decision-making. Users’ perspectives should be addressed and they should be involved in shared governance and guiding further science and policy-making.

## Supplementary information

Supplementary Table S1. Characteristics and reproductive decisions of participants (per couple, n=25)

Supplementary Appendix. Interview guide: explanations of technologies NIPD, GGE, SGE
