# Supplementary material for: How will new genetic technologies, such as gene editing, change reproductive decision-making? Views of high-risk couples
Source: Eur J Hum Genet. 2020 Aug 9;29(1):39–50. doi: 10.1038/s41431-020-00706-8 (PMC7852899; doi:10.1038/s41431-020-00706-8)
Supplement: Supplementary file 1 — Supplementary Table S1. Characteristics and reproductive decisions of participants (per couple, n=25) [file 41431_2020_706_MOESM1_ESM.docx]

**Supplementary Table S1. Characteristics and reproductive decisions of participants (per couple, n=25)**

# Manuscript: Van Dijke et al. How will new genetic technologies, such as gene editing, change reproductive decision-making? Views of high-risk couples

| # | Carrier (man/ woman/ couple) | Inheritance | Parents of (n) affected child(ren) | Parents of (n) healthy child(ren) | PGT  (yes (completed^a^/ ongoing/stopped)/no) | PND  (yes (CVS, AC, FSD)/no) | Planning to have (more) children  (yes/ maybe / no/ unknown) |
| --- | --- | --- | --- | --- | --- | --- | --- |
| 1. | Woman | AD |  | 1 | Yes (completed) | No | Maybe |
| 2. | Man | AD |  |  | Yes (ongoing) | No | Unknown |
| 3. | Woman | AD |  | 1 | Yes (completed) | No | Yes |
| 4. | Man | AD |  | 1 | Yes (ongoing) | Yes (CVS) | Unknown |
| 5. | Man | AD |  | 1 | No | Yes (AC) | Yes |
| 6. | Man | AD |  | 1 | Yes (stopped, due to spontaneous pregnancy) | No | Yes |
| 7. | Couple | AR | 1 | 1 | No | No | No |
| 8. | Couple | AR | 1 (deceased within first year) | 1 | Yes (completed) | Yes (CVS) | Maybe |
| 9. | Couple | AR | 1 |  | No | No | No |
| 10. | Couple | AR | 1 |  | Yes (ongoing) | Yes (AC) | Currently pregnant |
| 11. | Couple | AR | 1 (deceased within first year) | 2 | Yes (stopped, due to spontaneous pregnancy) | Yes (2x CVS) | No |
| 12 | Woman | XLR |  |  | Yes (stopped, relationship ended) | No | Yes |
| 13. | Woman | XLR | 1 | 1 | Yes (stopped, due to spontaneous pregnancy) | Yes (FSD) | No |
| 14. | Woman | XLR |  | 1 | No | Yes (CVS) | Maybe |
| 15. | Woman | XLR |  |  | Yes (ongoing) | No | Currently pregnant |
| 16. | Woman | XLR |  | 2 | No | Yes (2x FSD, followed by 1x CVS) | No |
| 17. | Woman | XLR |  | 1 | Yes (stopped, due to spontaneous pregnancy) | Yes (FSD) | Yes |
| 18. | Woman | XLR |  | 1 | Yes (stopped, did not succeed, a natural pregnancy occurred) | No | Yes |
| 19. | Woman | XLR |  | 2 | Yes (stopped due to spontaneous pregnancy) | Yes (2x CVS and 1x AC) | Currently pregnant |
| 20. | Woman | XLR |  | 1 | Yes (stopped, due to spontaneous pregnancy) | Yes (FSD and CVS) | No |
| 21. | Woman | XLD |  | 1 | Yes (stopped, due to spontaneous pregnancy) | Yes (CVS) | Maybe |
| 22. | Woman | XLD |  | 1 | No | Yes (CVS) | Maybe |
| 23. | Woman | XLD |  |  | Yes (ongoing) | No | Maybe |
| 24. | Woman | Chromosome imbalance |  | 1 | Yes (completed) | Yes (CVS) | Maybe |
| 25. | Man | Chromosome imbalance |  |  | Yes (stopped, did not succeed) | No | Yes |

^a^ Completed: the PGT process resulted in a pregnancy.

AD, Autosomal Dominant; AR, Autosomal Recessive; AC, Amniocentesis; CVS, Chorionic Villus Sampling; FSD, Fetal Sex Determination; PGT, Preimplantation Genetic Testing; PND, Prenatal Diagnosis; XLD, X- Linked Dominant; XLR, X- Linked Recessive.
