# Supplementary material for: How will new genetic technologies, such as gene editing, change reproductive decision-making? Views of high-risk couples
Source: Eur J Hum Genet. 2020 Aug 9;29(1):39–50. doi: 10.1038/s41431-020-00706-8 (PMC7852899; doi:10.1038/s41431-020-00706-8)
Supplement: Supplementary file 2 — Supplementary Appendix. Interview guide: explanations of technologies NIPD, GGE, SGE [file 41431_2020_706_MOESM2_ESM.docx]

# Supplementary Appendix. Interview guide: explanations of technologies NIPD, GGE, SGE

Manuscript: Van Dijke et al. How will new genetic technologies, such as gene editing, change reproductive decision-making? Views of high risk couples

**Text summary: Part of the interview guide that describes the background information and questions about the new technologies NIPD, GGE and SGE.**

**Introduction**

With this interview we want to explore which considerations and experiences are of importance to the reproductive decision-making process with regard to having children and the various options that are available (such as prenatal diagnosis or pre-implantation genetic testing (PGT)), for couples with an increased risk of having an affected child. In addition, I will ask questions on whether the availability of future genetic techniques would influence previous reproductive decision-making. With the results from these interviews, the offer of genetic reproductive technologies and counseling, can be aligned as closely as possible to the wishes and demands of those who use these technologies.

*The first part of the interview focuses on the previous reproductive decision-making process of couples. What decision they made and how they have come to those decisions.*

**New technologies**

[Continue interview with new techniques] Interviewer: Besides embryo selection/preimplantation genetic testing (PGT) and invasive prenatal diagnosis (PND) that we discussed in this interview, I would also like to talk to you about new technologies that are currently being developed. Some of these might become available in the (near) future, while others may never be safe enough to be implemented.

For our study, we would like to know how couples like yourself look at these future technologies and whether or not these technologies would affect couples’ reproductive decisions, and why. We would therefore like to know what your views are on the technologies that are currently available and on the new technologies that might become available in the (near) future. Your opinion is very important to us. Little is currently known about what people think of these new technologies. I am going to discuss three new technologies with you in a hypothetical way, emphasizing that none of them are currently available at the moment (in the Netherlands).

**Non-invasive prenatal diagnosis (NIPD):**

Have you ever heard of non-invasive prenatal diagnosis, also known as NIPD? [If people make the comparison with NIPT for fetal aneuploidy, the differences are explained]. If so, can you tell me what you know about this technique and how you became familiar with it?

Explanation: Suppose that with NIPD, your blood sample could be tested early in pregnancy, to safely and reliably test whether your unborn child has the condition.

Would you prefer to opt for NIPD instead of, for example, chorionic villus sampling? Why (not)? Suppose this method would be offered, would this be an option for you? Would the availability of this technique have influenced your earlier decisions (e.g. regarding PND/PGT)? Why (not)?

Do you have any doubts about this option? If so, can you elaborate?

**Germline gene editing (GGE):**

Have you ever heard of germline gene editing, also known as embryo modification? If so, can you tell me what you know about this technique and how you became familiar with it?

Explanation: With embryo modification it would be possible to genetically ‘repair’ the embryo, to ensure that your future child does not have the genetic condition that you are carrying. This technique is currently not available but might be an option in the future. A mutation in the DNA can cause a certain disease. Germ-line gene editing is a technique that would enable medical professionals to repair this mutation in the DNA of the oocytes/sperm or in the embryo. The process of GGE will possibly look similar to PGT.

What do you think about GGE? Can you describe your feelings? Why do you feel this way?
Would you possibly consider this option, if possible? Why (not)?

You have previously (not) opted for PGT – if it would be possible to genetically repair the embryos with the mutation, would you prefer that? Would you possibly consider this? Why (not)?
Do you have any doubts about this technique? If so, can you elaborate?

**Somatic gene editing (SGE):**

Have you ever heard of somatic gene editing or somatic gene therapy? If so, can you tell me what you know about this technique and how you became familiar with it?

Explanation: With somatic gene editing, body cells are repaired. The cells are removed from the person with the disorder and genetically modified/repaired in the laboratory. Under certain conditions, these cells can be multiplied and returned to the patient via the bloodstream. It might also be possible in the future to introduce the "healthy” or repaired cells directly into the body via an infusion. In this way, genetic conditions can be repaired and treated in the future, soon after birth. This technique is currently not available but might be an option in the future.

What do you think about SGE? Would you possibly consider this? Why (not)?

Do you have any doubts about this technique? If so, what?

Are there any of these techniques (just explained/discussed) that are not acceptable to you? Why? Which of all the techniques would you prefer most? Why?

Do you think that the three options mentioned above (i.e NIPD, germline gene editing or embryo modification and somatic gene editing) should be available for everyone, for certain groups or for no one? Why?

**Closing questions interview**

Thank you for participating in this research and for your sincerity.

- Do you have any questions at the moment?

- Do you want to add anything else?

- Have I forgotten something?

- Were certain questions difficult or unclear / unpleasant to answer?

- How did you like participating in this interview?

**Finally I will give you a form with a few short questions about your background, would you like to fill this in for me?**Age, education, ethnical background, belief and which conditions occur in your- or your partner’s family?
